# Impaired Clearance of Apoptotic Cells in Chronic Inflammatory Diseases: Therapeutic Implications

**DOI:** 10.3389/fimmu.2014.00354

**Published:** 2014-08-01

**Authors:** Zsuzsa Szondy, Éva Garabuczi, Gergely Joós, Gregory J. Tsay, Zsolt Sarang

**Affiliations:** ^1^Department of Dental Biochemistry, Faculty of Dentistry, University of Debrecen, Debrecen, Hungary; ^2^Department of Internal Medicine, Faculty of Medicine, Chung Shan Medical University Hospital, Taichung, Taiwan; ^3^Department of Biochemistry and Molecular Biology, Faculty of Medicine, University of Debrecen, Debrecen, Hungary

**Keywords:** apoptotic cell, phagocytosis, inflammation, autoimmunity, atherosclerosis, obesity, type 2 diabetes, therapy

## Abstract

In healthy individuals, billions of cells die by apoptosis every day. Removal of the dead cells by phagocytosis (a process called efferocytosis) must be efficient to prevent secondary necrosis and the consequent release of pro-inflammatory cell contents that damages the tissue environment and provokes autoimmunity. In addition, detection and removal of apoptotic cells generally induces an anti-inflammatory response. As a consequence improper clearance of apoptotic cells, being the result of either genetic anomalies and/or a persistent disease state, contributes to the establishment and progression of a number of human chronic inflammatory diseases such as autoimmune and neurological disorders, inflammatory lung diseases, obesity, type 2 diabetes, or atherosclerosis. During the past decade, our knowledge about the mechanism of efferocytosis has significantly increased, providing therapeutic targets through which impaired phagocytosis of apoptotic cells and the consequent inflammation could be influenced in these diseases.

## Introduction

Efficient execution of apoptotic cell death followed by efficient clearance mediated by professional and by non-professional neighboring phagocytes, is a key mechanism in maintaining tissue homeostasis. Every day, billions of our cells die and get cleared without initiating inflammation and an immune response (1). Proper clearance of dead cells also contributes to the initiation of tissue repair processes following injury (2–4). In addition, efficient removal of apoptotic neutrophils is also a key event in the resolution of inflammation (5).

Increasing evidence suggest that improper clearance of apoptotic cells, being the result of either genetic anomalies and/or a persistent disease state, contributes to the establishment and progression of a number of human diseases via affects on the maintenance of tissue homeostasis, tissue repair, and inflammation (6). Autoimmune disorders, in which both animal models and human research indicate a strong relationship between improper clearance and the development of the disease, represent the best characterized example of such diseases. The regulated nature of apoptotic cell death normally prevents the leakage of the immunogenic intracellular contents. If, however, these cells are not promptly cleared, they undergo secondary necrosis leading to the release of the intracellular antigens and DNA, which in the long-term provoke an auto-inflammatory response (7). Thus, in most of the knock out mice in which efferocytosis is impaired, systemic lupus erythematosus (SLE) like autoimmunity develops (8–13). Human SLE is also accompanied by improper efferocytosis (7), and can develop also as a result of a genetic deficiency of the phagocytosis process (13).

While in SLE improper clearance of apoptotic cells affects all the tissues, in several chronic inflammatory respiratory diseases, such as chronic obstructive pulmonary disease (COPD), cystic fibrosis, and asthma, increased numbers of apoptotic cells are seen only in the sputum and lung tissue (14). Though so far no evidence was provided for a definite linkage between genetic anomalies affecting efferocytosis and lung disease, inefficient apoptotic clearance in the lung was detected in all these respiratory diseases (15).

Macrophages play a key role in the development of atherosclerosis, and impaired clearance of apoptotic macrophages characterizes the late plaques, in which uncleared apoptotic cells undergo secondary necrosis leading to the formation of an unstable necrotic core and the maintenance of inflammation (16). Impaired efferocytosis, however, might also contribute to the development of the disease, as knock out mice deficient in efferocytosis are prone to develop atherosclerosis on LDL or ApoE null genetic backgrounds (17–20). An excess of apoptotic cells was detected in a numerous neurodegenerative diseases as well, such as Parkinson’s, Alzheimer’s, and Huntington’s disease (21). Though the elevated levels of apoptotic cells might also be the result of an increased neuronal cell death, in these diseases loss of signaling by fractalkine (an apoptotic cell “find me” signal) resulted in an increase in the number of dying cells and worsening of the disease (22).

Interestingly, type 2 diabetes and obesity were also shown to be associated with impaired phagocytosis of apoptotic β-cells in the pancreas in autoimmune diabetes-prone rats (23) and in ob/ob and db/db mice (24). The phenomenon seems to be related to an enhanced saturated and/or decreased ω-3 fatty acid composition of the plasma membrane, which leads to a decreased phosphatidylinositol 3-kinase activation during the uptake of apoptotic cells (24).

## Mechanisms Contributing to Efficient Phagocytosis of Apoptotic Cells

### “Find me” and “eat me” signals

**Figure 1:**
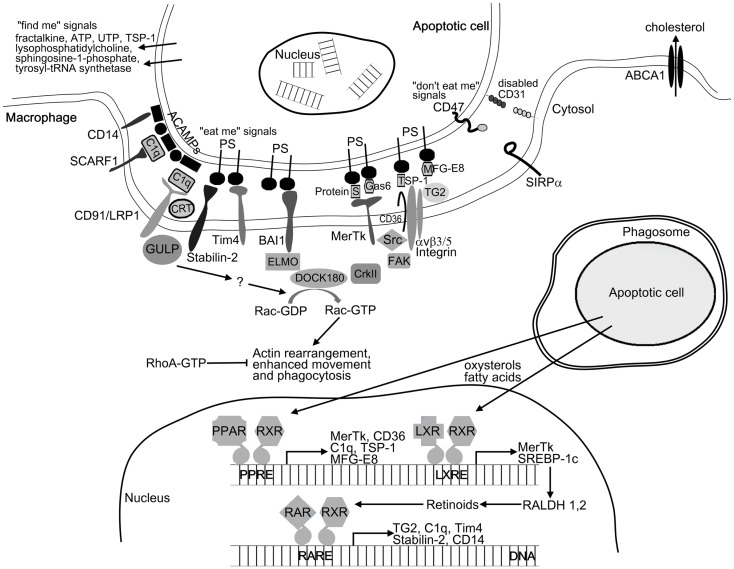
**Mechanism of apoptotic cell clearance**. For initiating phagocytosis apoptotic cells release “find me” signals for the phagocytes. After finding the recognition of apoptotic cells by phagocyte receptors is mediated by the display of “eat me” signals (e.g., PS and ACAMPs) and the disappearance of the so-called “do not eat me” signals (e.g., CD31 and CD47) on the apoptotic cell surface. Among others, these receptors include the PS receptors (Tim4, stabilin-2, and BAI1) and receptors such as MerTk, SCARF1, CD36, and integrin αvβ5 together with TG2 recognizing apoptotic cells through bridging molecules (e.g., TSP-1, C1q, Gas6, MFG-E8, and protein S). Binding of apoptotic cells to the phagocytic receptors triggers two evolutionary conserved signaling pathways. MerTk, BAI1, and αvβ3/5 receptors will activate the DOCK180/CrkII/ELMO complex, while CD91/LPR1 and stabilin-2 will activate the adaptor protein GULP. Both pathways converge on the small GTPase Rac, which initiates actin rearrangement and phagocytosis. Following engulfment, apoptotic cell derived lipids (oxysterols and fatty acids) trigger the lipid-sensing LXR and PPAR receptors leading to enhanced retinoid production. Retinoid receptors together with LXR and PPARs upregulate a number of phagocytic receptors to further enhance the engulfing capacity of macrophages under conditions when the rate of apoptosis is increased.

To ensure effective removal, apoptotic cells recruit phagocytes by releasing various soluble “find me” signals. These signals include lysophosphatidylcholine (25), CX3CL1/fractalkine (26), sphingosine-1-phosphate (27), the nucleotides ATP and UTP (28), thrombospondin-1 (TSP-1) (29), and cleaved human tyrosyl-tRNA synthetase (30). Upon arrival at the target cells, phagocytes must distinguish between apoptotic and viable cells. Apoptotic cells display apoptotic cell-associated molecular patterns (ACAMPs), which includes the appearance of “eat me” signals on their cell surface (5). These can bind either directly or through bridging molecules to receptors on phagocytes (Figure 1). Externalization of phosphatidylserine (PS) on the outer leaflet of the cell membrane is the best characterized “eat me” signal during apoptosis. The T-cell immunoglobulin- and mucin-domain-containing molecule (Tim4), stabilin-2, and brain-specific angiogenesis inhibitor 1 (BAI1) were reported to directly recognize PS on dying cells (31–33), while other receptors such as Mer tyrosine kinase (MerTk), scavenger receptor SCARF1, CD36, and integrin αv/β3/β5 together with CD36 or tissue transglutaminase (TG2) recognize apoptotic cells through bridging molecules. Gas6 and protein S were found to facilitate apoptotic cell clearance by recognizing PS on apoptotic cells and MerTk receptor on phagocytes (34, 35). TSP-1 and milk-fat globulin-E8 (MFG-E8) also bind to PS and are recognized by the integrin αv/β3/CD36 or integrin αv/β3/TG2 receptor complexes, respectively (36–38). The collectin family member serum protein C1q also serves as a bridging molecule by recognizing annexin A2 and A5 on the apoptotic cells (39) and binding either SCARF1 scavenger receptor or the calreticulin associated LRP1/CD91 receptor on phagocytes (39, 40). The LPS coreceptor CD14 can also act as a tethering receptor for apoptotic cells, albeit its exact ligand remains unknown (41). Distinguishing between apoptotic and viable cells is further ensured by the “do not eat me” signals, which inhibit the uptake of living cells. CD47, activating SIRPα receptor, is one of these signals being expressed on living cells but altered or diminished on apoptotic cell surface (42). Additionally, homophilic interaction between CD31 on the target cells and macrophages was shown to mediate cell detachment from phagocytes, thus inhibiting phagocytosis of living cells (43).

### Engulfment and ingestion of the apoptotic corpses

Uptake of the apoptotic cells requires the reorganization of the actin filament network, which drives the movement of the cell, formation of the phagocytic cup and the phagosome. This process is regulated by the small GTPases RhoA, Cdc42, and Rac. While RhoA activation was found to inhibit apoptotic cell phagocytosis, Cdc42, and Rac were shown to enhance it (44). Phagocytic receptors activate two evolutionary conserved pathways both converging on the activation of Rac-1, a small GTPase (45) (Figure 1). The first pathway is initiated by MerTk or integrin αv/β5 receptors (46, 47), resulting in association of the adaptor protein ELMO with the Rac GEF DOCK180 forming a bipartite GEF (48). Recruitment of the ELMO/DOCK180 complex to the cell membrane might require the adaptor protein CrkII, but binding of ELMO to the carboxyl terminus of BAI1 also recruits DOCK180 to the phagocytic membranes (33). The second pathway activating the Rac is initiated by LRP1 (CD91) (49) or by stabilin-2 receptors followed by recruitment of the adaptor protein GULP (50). Further steps, resulting in the activation of Rac are still unclear. The newly formed phagosome must fuse with lysosomes to degrade the dead cells. Recently, several autophagic genes were described to participate in phagosome maturation (51, 52). Following phagolysosomal fusion, lysosomal enzymes degrade the content of phagolysosomes. Lysosomal cathepsin protease CPL-1 was found to be indispensable in the digestion of apoptotic cell derived proteins (53), while lysosomal DNase II degrades the DNA content (54).

### Reprograming of phagocytes by apoptotic cell content

Engulfment of apoptotic cells delivers excess materials to the phagocytes. Some of these materials can be completely degraded, while the excess of non-digestible cholesterol is removed via ATP-binding cassette (ABC) transporters (Figure 1). Both PS (55) and lipid-sensing nuclear receptors (56, 57) can upregulate the levels of the ABCA1 transporter. The ingested macromolecules provide the extra energy required for prolonged phagocytosis. However, if too much energy is generated, engulfing cells upregulate the mitochondrial uncoupling protein 2 (UCP2) and dissipate H^+^ gradient to reduce mitochondrial membrane potential (58). UCP2 also decreases reactive oxygen species formation.

To ensure efficient long-term phagocytosis, apoptotic cells reprogram macrophages not only by altering their metabolism but also by increasing the expression of a number of phagocytic receptors via activating peroxisome proliferator-activated receptor (PPAR)δ/γ and liver X receptor (LXR)α/β receptors by their lipid content (59–61). This process is partially mediated via upregulation of endogenous retinoid synthesis (62, 63).

While the phagocytosis of a variety of pathogenic targets normally triggers a pro-inflammatory response in macrophages, ingestion of apoptotic cells by macrophages induces an anti-inflammatory phenotype. The earliest anti-inflammatory activity of the apoptotic cell is manifest as an immediate-early inhibition of macrophage pro-inflammatory cytokine gene transcription and is exerted directly upon binding to the macrophage (64). Subsequently, both nuclear receptors (65, 66) are activated and soluble mediators are released from macrophages, which act in a paracrine or autocrine fashion to amplify and sustain the anti-inflammatory response (67, 68). During the resolution of inflammation the reprogramed macrophages appear as pro-resolving CD11b^low^ macrophages (69) that express immunoregulatory 12/15-lipoxygenase (70) involved in the formation of pro-resolving lipid mediators, termination of phagocytosis, and emigration to lymphoid organs (69) required for the proper termination of the inflammatory program. This process is regulated by the expression of a typical chemokine receptor D6 on the surface of apoptotic neutrophils (71).

Since improper efferocytosis might contribute to both the initiation and the maintenance of human diseases, enhancing phagocytosis might provide a therapeutic possibility to influence the progression of these diseases.

## Therapeutic Possibilities for Enhancing Efferocytosis in Diseases in Which Clearance of Apoptotic Cells is Impaired

### Affecting recognition and binding of apoptotic cells

If lack of sufficient MFG-E8 production leading to improper efferocytosis participates in the pathomechanism of a disease, providing MFG-E8 in recombinant protein form to the site of acute inflammation might enhance the efficiency of efferocytosis. Indeed, a decreased MFG-E8 expression was found in inflamed colons during the acute phase of murine experimental colitis, and intrarectal treatment with recombinant MFG-E8 ameliorated colitis by reducing inflammation and improving disease parameters (72). Alternatively, both prolactin (73) and glucocorticoids (74) can enhance MFG-E8 production providing a theoretical possibility for enhancing its expression in macrophages systematically.

MFG-E8 contains a PS binding domain, as well as an arginine–glycine–aspartic acid (RGD) motif, which enables its binding to integrins. Opsonization of the apoptotic cells and binding to integrins on the surface of phagocytic cells, mediates the engulfment of the dead cell. Based on this observation, an RGD–anxA5 was designed, and it was shown that introduction of RGD transformed the annexin A5, a molecule that binds to PS of apoptotic cells, from an inhibitor into a stimulator of efferocytosis (75). While recombinant MFG-E8 or the RGD–anxA5 could be utilized in acute inflammation, long-term administration of MFG-E8 leads to obesity, because it stimulates the fatty acid uptake of adipocytes (76). It is an open question, whether chronic administration of RGD–anxA5 would have the same side effects.

While MFG-E8 acts as a bridging molecule for integrins, Gas6, and protein S are bridging molecules for MerTk. Thus in cases, where MerTk plays a driving role in efferocytosis, such as cardiac repair after myocardial infarction (4), provision of Gas6 or protein S could similarly accelerate phagocytosis of apoptotic cells and tissue repair. Glucocorticoids enhance phagocytosis by making efferocytosis MerTK dependent (77), thus combining glucocorticoids and Gas6 or protein S might have a synergistic effect.

Other bridging molecules, such as collectins, were also reported to promote efferocytosis. Macrolide antibiotics, which have wide-ranging anti-inflammatory effects, were found to enhance efferocytosis by enhancing the expression of collectins (78). The therapeutic potential of these drugs has already been recognized, as they are successfully used in the treatment of COPD, cystic fibrosis, or asthma (79).

### Targeting lipid-sensing nuclear receptors with the aim of increasing the expression of phagocytic receptors or their bridging molecules

Since nuclear receptor signaling is strongly associated with enhanced efferocytosis and suppression of inflammation, glucocorticoids, PPARγ, PPARδ, and LXR agonists or retinoids are logical therapeutic targets in diseases in which efferocytosis is impaired.

Glucocorticoids, the most widely used anti-inflammatory drugs, were shown to enhance phagocytosis of apoptotic cells by increasing the expression of the phospholipid binding protein annexin A1 and its receptor ALXR (6, 80), as well as that of MerTK (73, 81). Long-term effects of glucocorticoids were reported to be mediated by PPARγ (82).

LXR agonists were shown to be effective in the treatment of mouse models of atherosclerosis and inflammation. Thus, LXR agonists [hypocholamide, T0901317, GW3965, or *N*,*N*-dimethyl-3β-hydroxy-cholenamide (DMHCA)] lower the serum cholesterol, and inhibit the development of atherosclerosis in murine models of atherosclerosis (83), while GW3965 inhibits the expression of inflammatory mediators in cultured macrophages as well as during *in vivo* inflammation (84). In addition, ligation of LXR was shown to prevent the development of SLE like autoimmunity in lpr mice (61) and decrease the disease severity in Alzheimer disease (85).

While all LXR ligands are effective in enhancing efferocytosis, T0901317, and GW3965 have been reported to increase plasma and liver triglycerides in some mouse models (86). DMHCA, however, reduced atherosclerosis in apolipoprotein E-deficient mice without inducing hypertriglyceridemia and liver steatosis (87). Thus, developing new potent and effective LXR agonists without the undesirable side effects may be beneficial for clinical usage (88). In this aspect, it is worth noting that we found daidzein, which is a plant-derived diphenolic isoflavone present in a number of plants and herbs (89) and has LXR and PPARγ activating activity (90), to enhance efferocytosis efficiently. Daidzein, similar to LXR agonists (91) induced the expression of TG2, as well as decreased the mitochondrial membrane potential (92).

In addition to LXR agonists, PPARγ agonists were also shown to reduce the neutrophil numbers in rodent models of acute inflammation, such as asthma and COPD (93) and to increase efferocytosis and therapeutic efficacy in a mouse model of chronic granulomatosis (94). PPARγ and PPARδ agonists were also shown to attenuate disease severity in experimental autoimmune encephalomyelitis, a murine model of multiple sclerosis (95, 96).

Both RAR and RXR ligands promote efferocytosis, but their effect is more pronounced if both receptors are activated (63). The effect of *in vivo* all-*trans* retinoic acid (ATRA) treatment on the development of lupus nephritis has already been tested in both mouse models (97, 98) and humans (99). Lupus nephritis is a major cause of morbidity and mortality in patients with SLE (100). Long-term ATRA treatment in SLE-prone mice resulted in longer survival, significant reduction of proteinuria, renal pathological findings, and glomerular IgG deposits. In humans, it also reduced proteinuria.

### Affecting the Rac-1/RhoA balance

Since previous studies have shown that Rac activation is required, while RhoA activation is inhibitory for effective clearance of apoptotic cells (44), compounds that alter the Rac-1/RhoA balance, by either increasing the level of active Rac-1 or decreasing the levels and/or activity of RhoA/Rho kinase, would be potential candidates for use in therapy. Among the anti-inflammatory drugs glucocorticoids were shown to alter the Rac-1/RhoA balance in macrophages (101). Another molecule that was shown to affect the Rac-1/RhoA balance is lipoxin A4, which enhances phagocytosis via a protein kinase A-dependent manner (102). Though lipoxin A4 activates both Rac-2 and RhoA, its positive effect on efferocytosis suggests that the ultimate balance favors Rac activation. Lipoxins have already been shown to reduce inflammation and tissue damage in a variety of rodent models (103), and their levels are low in cystic fibrosis patients (104). In addition, exposure to daidzein also enhances Rac activity (92).

Statins are 3-hydroxy-3-methylglutaryl coenzyme A-reductase inhibitors with potent anti-inflammatory effects, largely due to their ability to inhibit the prenylation of Rho GTPases, including Rac-1 and RhoA. Since proper membrane localization of these proteins determines their function, statins inhibit the effectiveness of G protein signaling. Lovastatin was shown to enhance efferocytosis *in vitro* both in naïve murine lung and in alveolar macrophages taken from COPD patients (105). It was demonstrated that its effect is related to a disproportional deactivation of the RhoGTPases favoring the activity of Rac-1, as well as to the activation of PPARγ (106).

During inflammation oxidant-mediated activation of RhoA and inhibition of efferocytosis might be reversed by antioxidant treatment. Thus, in an LPS-induced lung injury model, antioxidants enhanced efferocytosis and reduced inflammation by inhibiting RhoA activation (107).

### Affecting phagosome maturation

Increasing evidence suggests that autophagy and phagocytosis processes are interactive and co-regulated. Thus, activation of autophagy during salivary gland cell death in the *Drosophila* requires the engulfment receptor Draper (108). In addition, association of LC3 with intracellular membranes described originally during autophagy was observed during phagocytosis as well (109). In line with these observations, oridonin, an active diterpenoid isolated from *Rabdosia rubesens*, was able to induce both autophagy and enhance efferocytosis in the human macrophage-like U937 cells. Moreover, enhancing autophagy by rapamycin also enhanced phagocytosis of apoptotic cells by U937 cells (110). Thus, autophagy inducers might also promote efferocytosis. Though rapamycin and the so-called rapalogs are the most effective clinically used inducers of autophagy, they have severe immunosuppressive effects (111). That is why alternative, non-toxic autophagy inducers (such as rilmenidine or carbamazepine) are being characterized for their pharmacological profile in suitable preclinical models (112, 113). In addition, other non-toxic compounds, such as resveratrol and spermidine, are also being evaluated for their potential to induce autophagy *in vivo* (114, 115). These two latter compounds were shown to induce autophagy by distinct pathways converging on the acetylproteome (116). Resveratrol was suggested to mediate the cardioprotective effect of red wine (117), while spermidine was shown to prolong the life span of various organisms in an autophagy-dependent manner (114). Though the effect of the latter compounds on efferocytosis has not been tested yet, it is interesting to speculate whether enhanced efferocytosis contributes to their observed beneficial *in vivo* effects.

### Altering the membrane lipid composition of macrophages

Finally, studies on ob/ob and db/db mice indicate that in type 2 diabetes, obesity, or atherosclerosis impaired efferocytosis might be related to altered membrane lipid compositions of macrophages. In these cases, fish oil diet had a reversal effect (24). ω-3 fatty acids provided by fish oil are known substrates for the biosynthesis of pro-resolving mediators, such as resolvins, protectins, and maresin which, similar to glucocorticoids or opsonization of apoptotic cells by iC3b (69, 118), act as enhancers of efferocytosis as well as promote the formation of CD11b^low^ macrophages (119).

## Concluding Remarks

Apoptotic cell death is an integral part of the cell turnover in many tissues. If, however, dead cells are not properly cleared, their content is released and induces tissue damage, as well as long-term inflammation. It is increasingly recognized that improper phagocytosis of apoptotic cells contributes to the establishment and progression of a number of human chronic inflammatory diseases. During the past decade, our knowledge about the mechanisms involved in efferocytosis increased significantly providing potential pharmacological targets through which the efficiency of apoptotic clearance could be increased. Since enhanced phagocytosis is coupled to an enhanced anti-inflammatory response, targeting efferocytosis might provide an additional possibility in the treatment of a numerous human chronic inflammatory diseases.

## Conflict of Interest Statement

The authors declare that the research was conducted in the absence of any commercial or financial relationships that could be construed as a potential conflict of interest.
